# Noise as a mechanism of anomalous face processing among persons with Schizophrenia

**DOI:** 10.3389/fpsyg.2013.00401

**Published:** 2013-07-16

**Authors:** Bruce K. Christensen, Justine M. Y. Spencer, Jelena P. King, Allison B. Sekuler, Patrick J. Bennett

**Affiliations:** ^1^Schizophrenia Research Unit, Department of Psychiatry and Behavioural Neuroscience, McMaster UniversityHamilton, ON, Canada; ^2^Department of Psychology, Neuroscience and Behaviour, McMaster UniversityHamilton, ON, Canada

**Keywords:** Schizophrenia, face perception, internal noise, calculation efficiency, face orientation

## Abstract

There is substantial evidence that people with Schizophrenia (SCZ) have altered visual perception and cognition, including impaired face processing. However, the mechanism(s) underlying this observation are not yet known. Eye movement studies have found that people with SCZ do not direct their gaze to the most informative regions of the face (e.g., the eyes). This suggests that SCZ patients may be less able to extract the most relevant face information and therefore have decreased calculation efficiency. In addition, research with non-face stimuli indicates that SCZ is associated with increased levels of internal noise. Importantly, both calculation efficiency and internal noise have been shown to underpin face perception among healthy observers. Therefore, the current study applies noise masking to upright and inverted faces to determine if face processing deficits among those with SCZ are the result of changes in calculation efficiency, internal noise, or both. Consistent with previous results, SCZ participants exhibited higher contrast thresholds in order to identify masked target faces. However, higher thresholds were associated with increases in internal noise but unrelated to changes in calculation efficiency. These results suggest that SCZ-related face processing deficits are the result of a decreased noise-to-signal ratio. The source of increased processing noise among these patients is unclear, but may emanate from abnormal neural dynamics.

## Introduction

People with Schizophrenia (SCZ) are impaired in recognizing and discriminating human faces in both upright and inverted orientations (Archer et al., 1992; Whittaker et al., 2001; Sachs et al., 2004). Moreover, although SCZ participants demonstrate visual processing deficits across a broad assortment of stimuli, face stimuli may be particularly problematic in this regard. For example, in a study by Chen et al. (2008), patients with SCZ were less accurate when locating or matching line drawings of faces compared to similar drawings of trees. This suggests that the ability to detect faces, compared to other visual stimuli, may be disproportionately impaired in this patient population^1^. Furthermore, when detecting trees, both patients with SCZ and healthy participants displayed similar stimulus inversion effects. However, stimulus inversion effects for faces were observed to be significantly reduced in people with SCZ^2^. The reduced face inversion effect observed in the patient population, in addition to the lack of an inversion effect when detecting trees, suggests an impairment that is particular to face detection in SCZ. Moreover, SCZ-related deficits in face discrimination increased significantly as the duration of the interval between the initial and target faces increased, suggesting that face deficits may exist in working memory, as well as perception (Chen et al., 2009). These results, and others (Novic et al., 1984; Walker et al., 1984; Phillips and David, 1995; Gur et al., 2002) establish that SCZ participants are impaired when processing faces and suggest that this deficit is not likely to be fully accounted for by a general visual processing impairment across all classes of objects (e.g., trees).

Moreover, it is broadly accepted that faces convey an enormous range of socially relevant information about one's identity, gender, age, ethnicity, mood, attractiveness, level of interest, current focus of attention, and/or intentions (Haxby et al., 2002; Little et al., 2011). In turn, this information influences an array of social phenomenon including social categorization (Quinn and Macrae, 2011), discrimination, stereotyping and prejudice (Quinn et al., 2003), judgment of others' emotional state and empathy (Freitas-Magalhães, 2011; Eisenberger, 2012), and romantic attraction, attachment, and friendship (Gobbini et al., 2004; Wang et al., 2010). These observations are relevant to the investigation of SCZ since patients with this disorder also demonstrate prominent social deficits (Penn et al., 1997; Tulloch et al., 2006) and many researchers have directly linked SCZ-related alterations in face processing with social impairment (e.g., Marwick and Hall, 2008). Consequently, investigations aimed at understanding the mechanisms of altered face processing also hold the promise of elucidating determinants of social dysfunction among in persons with SCZ.

However, despite reliable evidence of impaired face processing among people with SCZ, few studies have assessed the mechanisms potentially responsible for this finding. A common speculation is that impaired face processing is a result of compromised neural functioning in the fusiform face area (FFA), or Brodmanns area 37. This suggestion is based on previous findings demonstrating reduced volume of the fusiform gyrus compared to other brain regions (Highley et al., 1999; Lee et al., 2002; Onitsuka et al., 2003). Additionally, reduced fusiform volume has shown to be associated with facial emotion recognition deficits in the population (Goghari et al., 2011). However, functional activation (as ascertained by fMRI) of the FFA has been indistinguishable between healthy and SCZ participants when performing face discrimination tasks (e.g., Yoon et al., 2006). These findings led Yoon et al. (2006) to suggest that other cortical mechanisms are likely responsible for impaired face processing in SCZ.

### Calculation efficiency and internal noise

Signal detection theory (SDT) (Green and Swets, 1966) provides an alternative way of conceptualizing how face perception differs between healthy individuals and those with SCZ. According to SDT, observers make responses by comparing an internal response evoked by a stimulus to a decision criterion. In classic formulations of SDT, the internal response is unidimensional. However, it is not the case that SDT applies only to stimuli that vary along a single dimension (e.g., tones that vary in intensity); in fact, the theory has been applied successfully in many contexts using multidimensional stimuli (Swets, 1996). SDT casts an internal response as an abstract decision variable rather than a direct response to a simple stimulus attribute like intensity. According to this idea, the decision variable is an index of the information relevant to a particular decision (e.g., a stimulus is or is not present; the face belongs to person A or person B) that may be calculated from several stimulus attributes. The nature of this calculation—which stimulus attributes are encoded, and how they are distilled into a single decision variable—influences the amount of information conveyed by the decision variable and, therefore, constrains performance in perceptual tasks. In the current study, the degree to which a decision variable calculation captures the available stimulus information will be referred to as calculation efficiency. In the case of face identification, a great deal of information is concentrated near the eyes and eye brows (Sekuler et al., 2004; Gaspar, 2006; Keil, 2008), and therefore an efficient calculation would utilize a decision variable based on the distribution of contours in those regions. An inefficient calculation, on the other hand, would derive a decision variable based on subtle changes in contrast from other less informative face areas (e.g., the forehead). Under this assumption, a proficient way to enhance the signal related to a target face (i.e., increase discrimination) is to use perceptual information from that face that best characterizes its uniqueness. Hence, one possible explanation for face perception deficits in people with SCZ is that they base their decisions on less informative aspects of faces (i.e., they have lower calculation efficiency). Evidence consistent with this idea has been described by Williams et al. (1999), who measured the eye movements of SCZ and healthy participants while they viewed human faces. Compared with healthy subjects, people with SCZ fixated less on the most informative regions of the face.

A second fundamental assumption in SDT is that internal responses are probabilistic: the internal response to an identical stimulus will vary across multiple presentations of the same stimulus. From a visual processing point of view, this internal variation, or noise, may arise from a variety of sources, such as jitter in eye position, fluctuations in attentiveness, or random fluctuations in the responses of sensory neurons. Obviously, internal noise degrades perceptual representations and limits performance in perceptual tasks. Hence, within the framework of SDT, poorer face perception in people with SCZ might be caused by elevated internal noise in face-processing mechanisms (Winterer and Weinberger, 2000; Rolls et al., 2008).

### Noise masking functions

How can we estimate internal noise and calculation efficiency? In this section we describe a psychophysical framework for estimating these quantities. Consider a simple face discrimination task: on each trial a subject is shown one of two faces and must decide which face was presented. Across trials, face contrast is varied to estimate a discrimination threshold (i.e., the contrast necessary for an observer to respond correctly on 70% of the trials). Finally, in this hypothetical experiment we will measure discrimination thresholds for faces embedded in *external noise* (i.e., a noise mask). Specifically, a zero-mean random number is added to the brightness, or contrast, at each pixel in the visual display. If the noise is independent at each pixel, then the noise is said to be “white.” Moreover, if the random numbers are selected from a Gaussian distribution, then the noise is said to be Gaussian and the strength of the noise is related to the variance of the distribution. Our hypothetical experiment will measure discrimination thresholds with white Gaussian noise that varies in strength. The resulting threshold-vs.-noise curve is referred to as a *noise masking function*.

**Figure 1:**
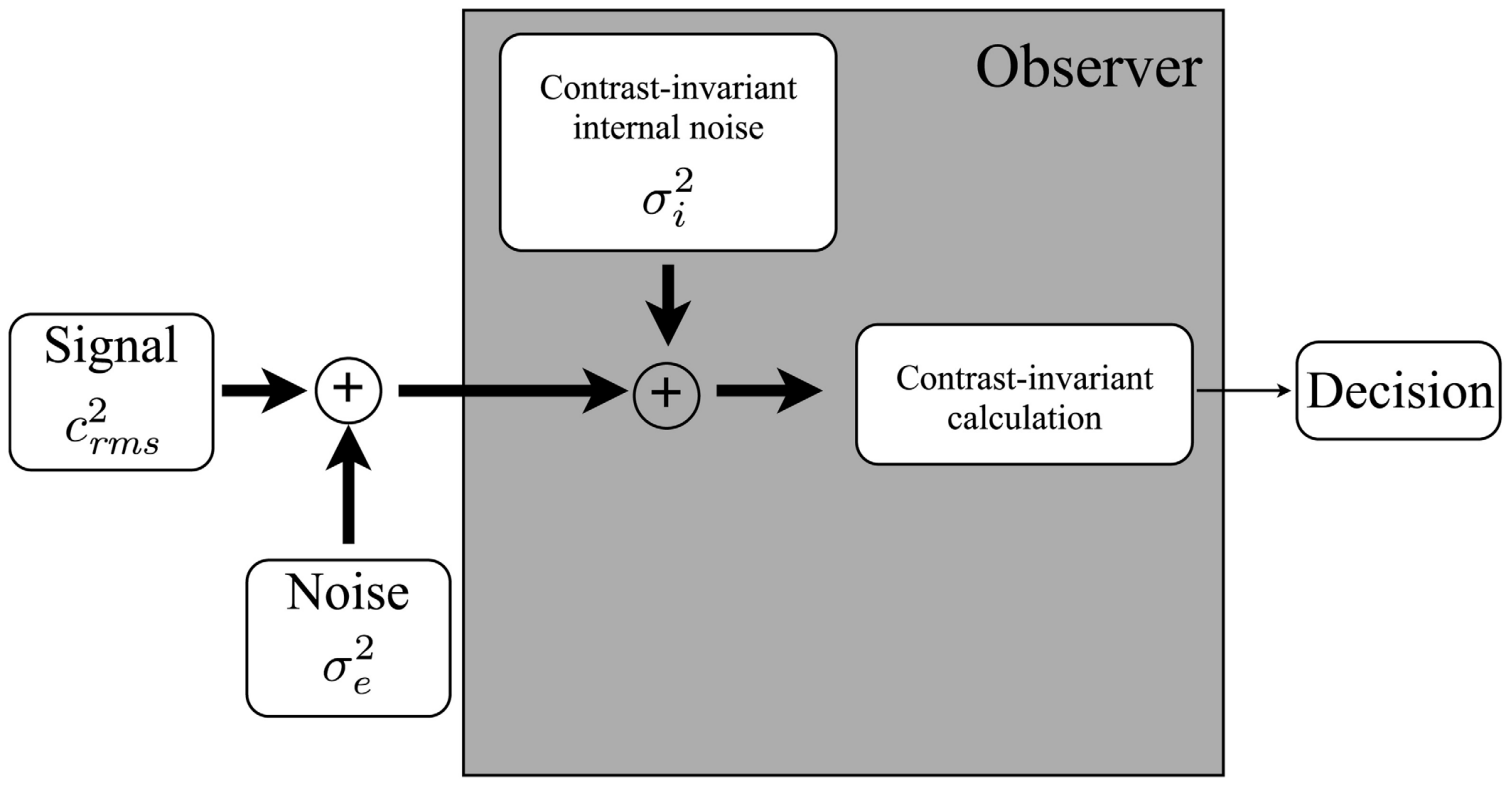
**A model of a human observer in a perceptual discrimination task**. The observer (i) transduces a noisy external stimulus; (ii) adds an internally-generated noise; (iii) applies a calculation that transforms the internal representation into a decision variable; and (iv) uses the decision variable to make a decision. The variance of the internal noise and the nature of the calculation are assumed to be independent of the contrast of the input. Adapted from Pelli (1981).

Pelli (1981) outlined a simple framework for interpreting noise masking functions (Figure 1): an observer receives a physical stimulus—in our case, a face embedded in external noise—which is transformed into an internal representation. An internal noise is added to the internal representation, and a calculation is performed that converts the internal signal-plus-noise variable into a decision variable. The variance of the internal noise and the nature of the calculation are assumed to be independent of stimulus contrast. Given this framework, discrimination threshold is related to the strength of the internal noise by the equation:

(1) $$c_{\text{rms}}^{2}=k(\sigma_{e}^{2}+\sigma_{i}^{2})$$

where *c*^2^_rms_ is threshold expressed as the squared rms contrast, or contrast variance, σ^2^_*e*_ is the variance of the external noise, and *k* and σ^2^_*i*_ are free parameters. The parameter σ^2^_*i*_ often is referred to as equivalent input noise because it is equal to the variance of the external noise that must be added to the stimulus to double the threshold relative to a no-noise baseline condition. In this framework, the threshold doubles when σ^2^_*i*_ equals σ^2^_*e*_, and therefore the equivalent input noise can be used as an estimate of the level of internal noise. Parameter *k* indicates the rate at which the threshold increases with increasing external noise, assuming internal noise remains constant, and is related inversely to the efficiency of the observer's internal calculation. The values of *k* and σ^2^_*i*_ are thought to reflect the influence of different processes and, therefore, measuring noise masking functions, as opposed to measuring a single threshold, provides more information about the processes that constrain perception.

**Figure 2:**
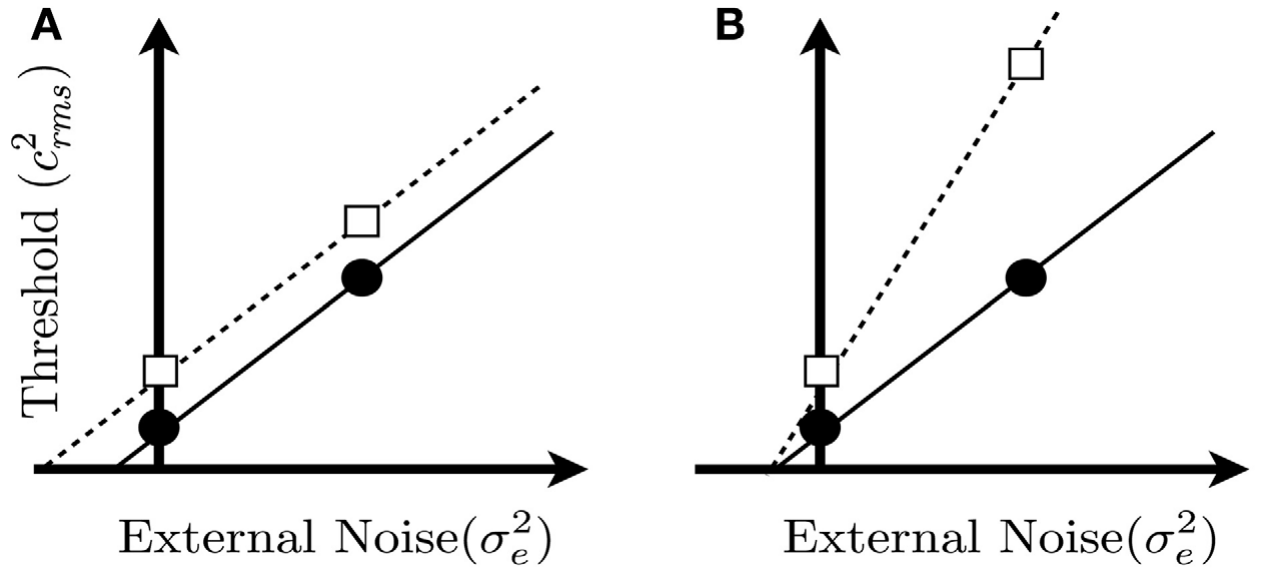
**Hypothetical noise masking functions predicted from Equation 1**. Each panel shows two noise masking functions for two groups, in which threshold is plotted as a function of the variance of the external noise mask. The two functions in each panel could represent thresholds measured from two groups of subjects, or under two different viewing conditions. The external noise is zero at the point where the x and y axes intersect. Panel **(A)** shows two masking functions that differ only in terms of equivalent input noise (i.e., parameter σ^2^_*i*_ in Equation 1). Panel **(B)** shows two masking functions that differ only in terms of calculation efficiency, which is related to the reciprocal of parameter *k* in Equation 1. Note that thresholds in the zero noise conditions, represented by the square and circle, are the same in the two panels. Hence, a threshold difference measured in the no-noise condition could be produced by a change in internal noise or calculation efficiency (or a combination of both factors).

Equation 1 predicts that threshold, expressed as *c*^2^_rms_, should be a linear function of the external noise variance. Figure 2 illustrates the effects of the parameters σ^2^_*i*_ and *k* on the masking functions predicted by Equation 1. In Figure 2A, the two masking functions differ only in terms of σ^2^_*i*_, and the resulting noise masking curves have the same slope but have different x-axis intercepts. In Figure 2B, the masking functions differ only in terms of *k*, and the resulting curves have the same x-axis intercepts but differ in terms of slope. Thresholds from a wide variety of tasks (Legge et al., 1987; Tjan et al., 1995; Dosher and Lu, 1998, 2000; Bennett et al., 1999; Gold et al., 1999, 2004, 2005; Pelli and Farell, 1999; Betts et al., 2007), including the discrimination of upright and inverted faces (Gaspar et al., 2008) are consistent with the predictions of Equation 1, including their linear relationship with masking noise.

### Objectives of the current study

Previous studies have shown that face perception is impaired in persons with SCZ. It is unclear, however, if these group differences in performance reflect differences in internal noise, calculation efficiency, or both factors. If the two groups differed primarily in terms of internal noise, then we would expect the noise masking functions in the two groups to differ like those shown in Figure 2A (i.e., discrepant intercept parameters). On the other hand, if the two groups differed primarily in terms of calculation efficiency, then the masking functions for the groups should differ like those shown in Figure 2B (i.e., discrepant slope parameters). To investigate these issues, the current experiment measured noise masking functions with upright and inverted faces in both healthy subjects and those with SCZ.

## Materials and methods

### Participants

Twenty-three people with SCZ (11 females, 12 males) and 24 healthy participants (12 females, 12 males) participated in this study. All patients met criteria for SCZ, Schizoaffective Disorder, or Schizophreniform Disorder, as confirmed by the Structured Clinical Interview for DSM-IV Axis I Disorders (SCID I) (First et al., 2001), but did not meet criteria for any other Axis I disorders, which was an exclusionary criterion. Healthy controls did not meet diagnostic criteria for any Axis I disorders. Structured clinical interviews were administered by either senior graduate students or research assistants, all of whom had received formal training in diagnostic interviewing. All participants had no self-reported history of neurological illness, brain injury, learning disability, current or past substance dependence, or medical conditions which could affect cognitive performance (e.g., coronary heart disease, type I diabetes). Participants also were excluded if they were taking psychotropic medication with known cognitive affects, including tricyclic antidepressants, anticholinergics, or benzodiazepines. Control participants were excluded if they reported having a first-degree relative with a SCZ-spectrum illness. Group means for healthy and patient participants were equivalent on age, years of education, an estimated Full Scale Intelligence Quotient (FSIQ) from the Wechsler Adult Intelligence Scale (Matrix Reasoning and Information subtests), 3rd Edition (Wechsler, 1997) and Wide-Range Achievement Test-III Reading subtest (WRAT-III; Wilkinson, 1993). In contrast, patients scored significantly lower the Hopkins Verbal Learning Test (HVLT) compared to controls (Brandt and Benedict, 2001). Patients with SCZ were administered the Positive and Negative Syndrome Scale (PANSS; Kay et al., 1987). In addition, both patients and controls were administered several scales from the Personality Assessment Inventory (PAI), including the Depression (Dep), Alcohol Problems (Alc), Drug Problems (Drg), Positive Impression Management (PIM), and Negative Impression Management (NIM) scales (Morey, 1991). Patients demonstrated significantly higher scores on the Dep, Alc, Drg, and NIM scales, but a significantly lower score on the PIM scale. Mean scale indices, however, were all within normal limits. Additionally, no single subject scored in a range suggesting deliberate distortion of their responses across both validity scales (i.e., PIM and NIM). All patients were medicated on a single antipsychotic agent [mean chlorpromazine = 416.67 mg (*SD* = 267.86)]. Patients' mean number of years since diagnosis was 8.04 (*SD* = 8.05) ranging from 1 year to 29 years. Table 1 provides information characterizing the study participants.

**Table 1:** **Means (SD) for demographic, neuropsychological, and clinical characteristics of the sample**.

**Variables**	**Initial**		**Upright condition**		**Inverted condition**	
	**Control (*n* = 24)**	**Patient (*n* = 23)**	**Control (*n* = 20)**	**Patient (*n* = 20)**	**Control (*n* = 19)**	**Patient (*n* = 17)**
**DEMOGRAPHIC**						
Age (years)	36.13 (12.08)	33.17 (8.50)	35.4 (11.75)	33.15 (8.22)	33.95 (12.03)	33.12 (9.10)
Education (years)	14.62 (2.48)	14.87 (2.47)	14.65 (2.56)	14.75 (2.38)	14.21 (2.32)	15.06 (2.44)
**NEUROPSYCHOLOGICAL**						
Estimated FSIQ	113.17 (14.30)	110.30 (14.35)	114.4 (13.62)	110.3 (14.16)	112.42 (15.39)	112.65 (13.22)
WRAT-3 Reading	104.37 (10.19)	103.87 (7.31)	104.9 (9.90)	103.15 (7.51)	103.58 (10.54)	104.24 (7.24)
HVLT-R	46.33 (5.29)	35.61 (11.77)^*^	46.15 (5.64)	34.95 (10.89)^*^	45.95 (5.76)	36.00 (11.52)^*^
**CLINICAL**						
PAI-Dep	44.41 (7.21)	57.04 (12.04)^*^	43.45 (6.35)	58.5 (13.05)^*^	44.16 (7.59)	59.24 (12.67)^*^
PAI-Alc	45.50 (3.56)	48.14 (6.06)^*^	44.95 (3.47)	48.35 (6.36)^*^	45.53 (3.66)	46.82 (5.58)
PAI-Drg	46.54 (5.52)	50.27 (8.29)^*^	46.70 (5.36)	50.7 (8.57)^*^	47.67 (5.62)	49.29 (6.48)
PAI-PIM	56.98 (6.01)	48.95 (10.91)^*^	58.10 (6.81)	48.75 (10.98)^*^	58.68 (6.23)	46.18 (9.11)^*^
PAI-NIM	46.52 (5.06)	59.27 (13.32)^*^	45.85 (3.13)	61.95 (16.61)^*^	46.16 (5.17)	62.53 (15.48)^*^
PANSS-Pos	−	40.87 (7.83)	−	40.45 (8.24)	−	41.00 (8.30)
PANSS-Neg	−	35.65 (6.48)	−	35.95 (6.76)	−	35.00 (6.75)

* *Indicates significant (p < 0.05) difference between controls and patients*.

*WRAT-3, Wide Range Achievement Test; HVLT-R, Hopkins Verbal Learning Test Revised; PANSS, Positive and Negative Symptom Scale*.

### Stimului and apparatus

Stimuli were generated by a Macintosh G4 computer and displayed on a CRT monitor using MATLAB and the Psychophysics Toolbox (Brainard, 1997; Pelli, 1997). The display had a frame rate of 85 Hz (non-interlaced) and a spatial resolution of 800 × 600 pixels, which from the viewing distance of 114 cm subtended 14.3° horizontally and 10.7° vertically. Face stimuli were based on digitized photographs of 10 frontal-view faces (5 males and 5 females), cropped to an oval window (width = 2.5°; height = 3.39°) that excluded areas showing chin, ears, and hair (see Gold et al., 1999 for details).

**Figure 3:**
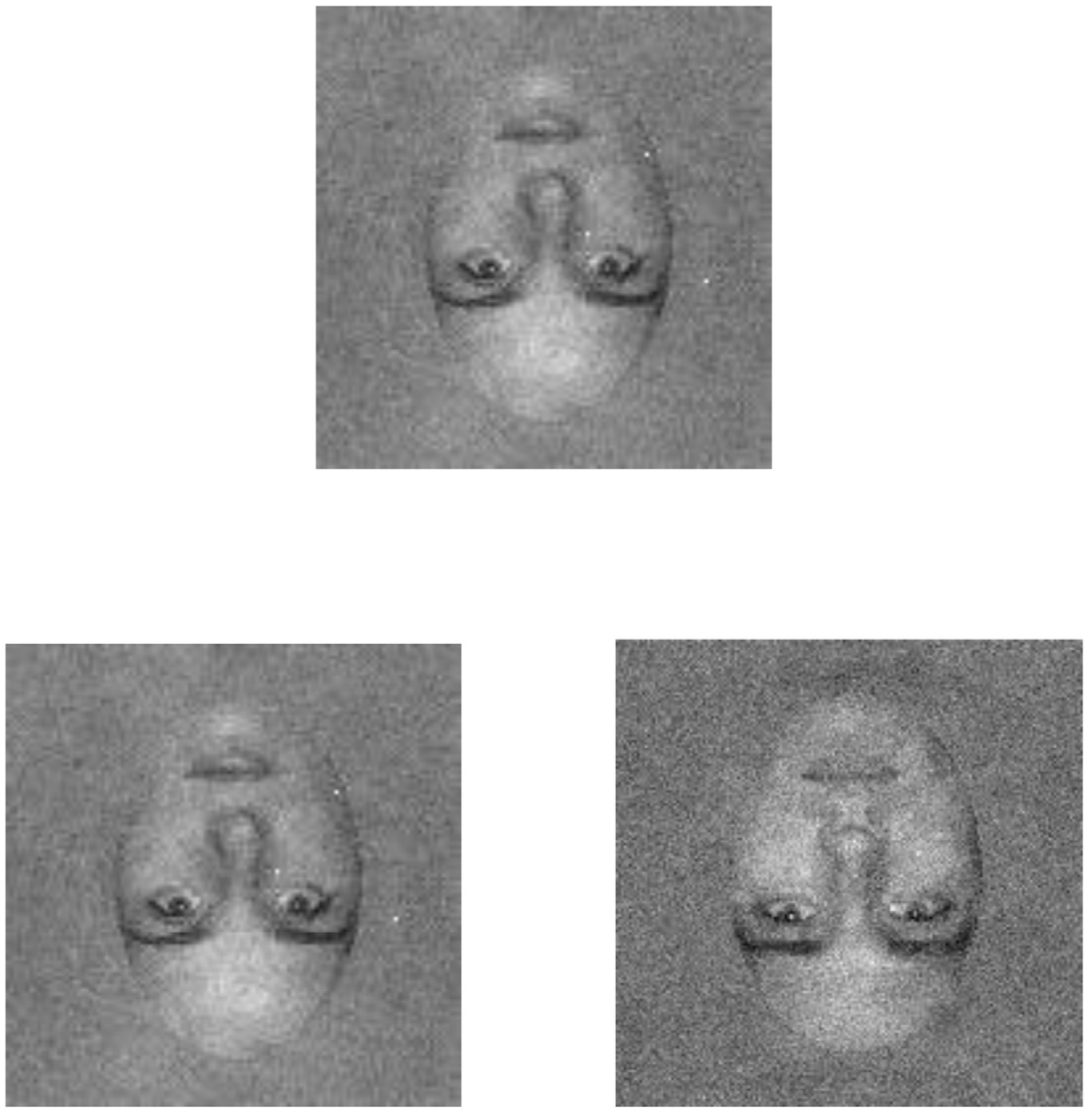
**An example of the (inverted) face stimuli as presented in the current experiment**. The inverted face is shown in both low (left) and high (right) external noise conditions. Participants were shown a target face (top) that was presented without noise. Following an inter-stimulus interval of 200 ms in which a static mask was presented, a pair of test faces consisting of the target and a distractor appeared. Participants were asked to discriminate the target face.

Thresholds were measured with a match-to-sample task. On each trial, two faces were selected randomly from the set of ten faces: one face was designated as the target, and the other as the distractor. A trial began with the presentation of a fixation cross at the center of the display. After a delay of 1 s, a noise-free version of the target face (rms contrast = 0.08) was presented for 200 ms centered at a location that was 2.29° above the fixation cross, and was followed by a high-contrast, static white noise mask that lasted for 200 ms. The offset of the mask was followed immediately by a 200 ms presentation of a pair of test faces—consisting of the target and distractor faces—centered 0.27° below and 2.29° to the left and right of the fixation cross. The target face could appear on the left or right with equal probability. The test faces were followed by the presentation (200 ms) of two high-contrast, static, white noise masks centered on the test faces (see Figure 3 for an example of the experimental stimuli). The participant's task was to determine which one of the two test faces was the target face, and auditory feedback was provided after each trial to indicate correct and incorrect responses.

The contrast of the test faces was varied across trials using a 3-down/1-up staircase procedure to estimate face identification threshold, which was defined as the contrast necessary to achieve a correct response rate of 79%. A staircase ended after 12 reversals, and a threshold was calculated by taking the average of the last eight reversals. In separate blocks of trials, thresholds were measured with upright and inverted faces. Within each block of trials, thresholds were measured in a low-noise condition, in which test faces were presented without noise, and a high-noise condition, in which faces were embedded in static white noise that had a contrast variance of 0.04. In the high-noise condition, a different noise field was computed for each test face on every trial. Note that in both conditions the test faces were *followed* by the presentation of high-contrast noise masks. Trials in the low- and high-noise conditions were intermixed randomly. Subjects were given 10 practice trials in each condition prior to starting the experiment.

## Results

Statistical analyses were performed with R (version 2.8.1; R Development Core Team, 2007). Effect size was expressed as Cohen's *f* (Cohen, 1988) using formulae described by Kirk (1995). For ANOVA tests in which *F* < 1, the effect size was assumed to be zero (see Kirk, 1995, p. 180).

**Figure 4:**
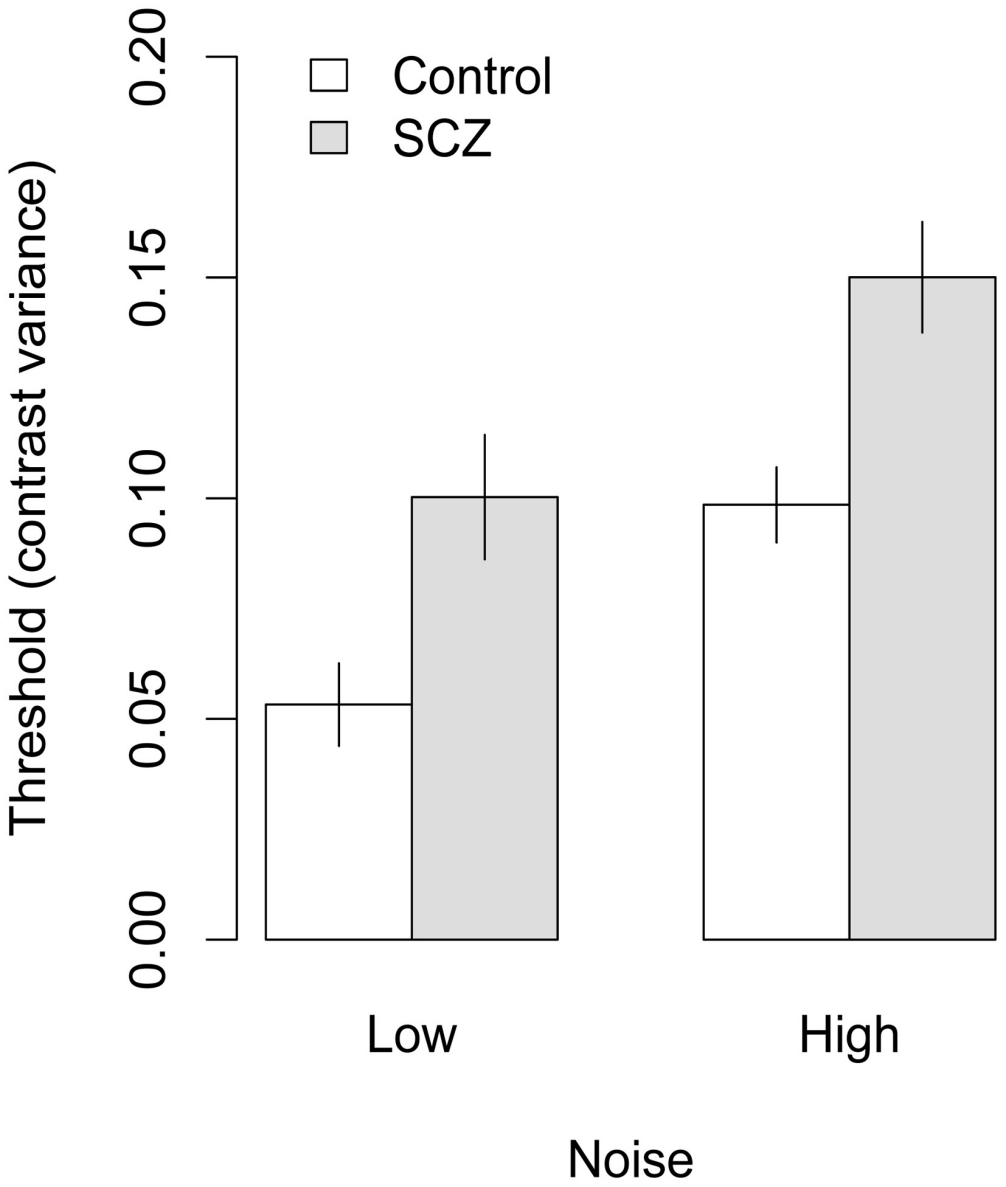
**Face identification thresholds measured with upright faces and expressed in terms of contrast variance**. In both high and low noise conditions, people with SCZ demonstrate higher thresholds, and thus, decreased performance for face identification. Error bars represent ±1 standard error.

**Figure 5:**
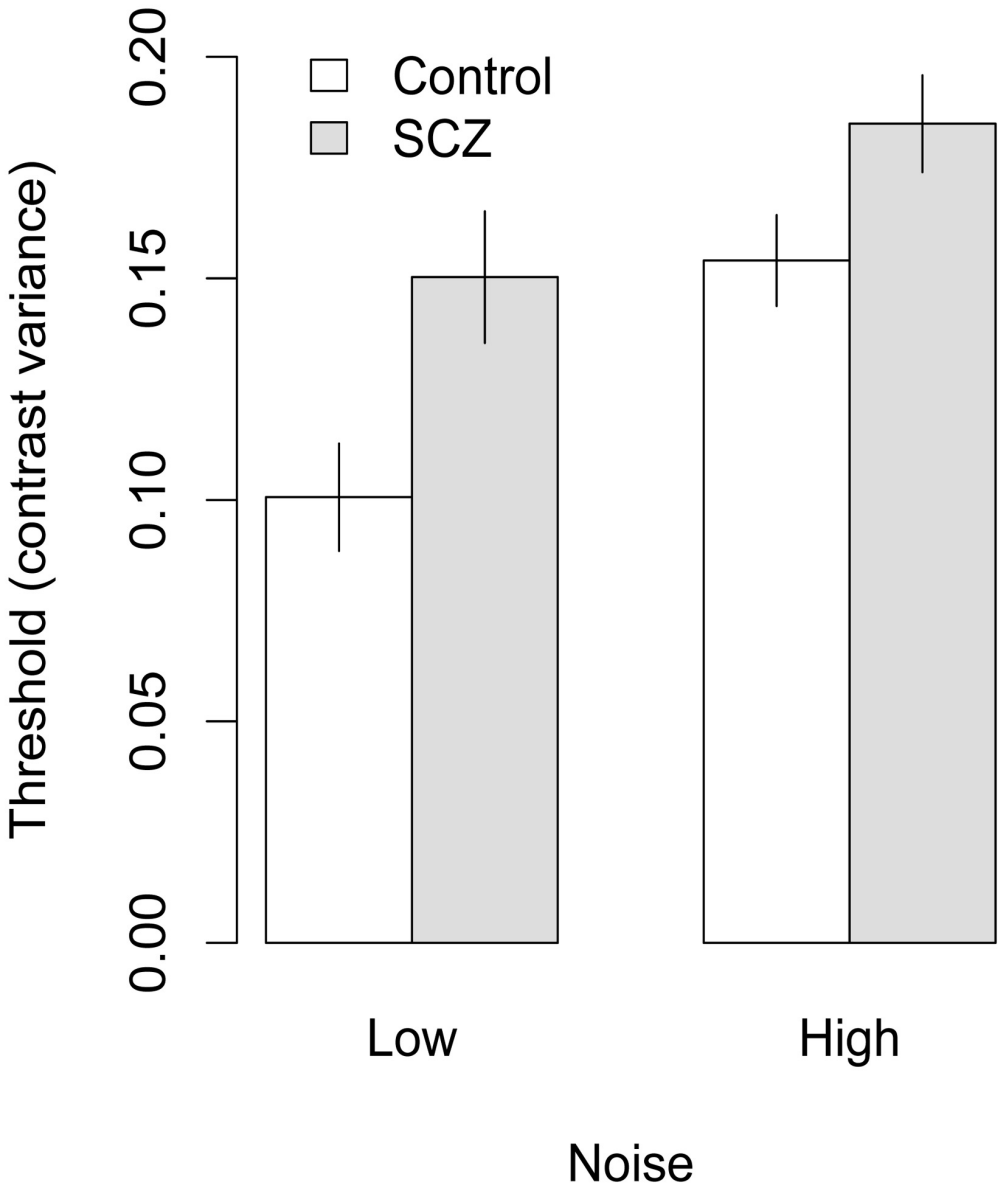
**Face identification thresholds measured with inverted faces and expressed in terms contrast variance**. In both high and low noise conditions, people with SCZ demonstrate higher thresholds, and thus, decreased performance for inverted face identification. Error bars represent ±1 standard error.

Face identification thresholds measured with upright and inverted faces, expressed in terms contrast variance, are shown in Figures 4 and 5, respectively. Thresholds were submitted to a 2 (group) × 2 (orientation) × 2 (external noise) analysis of variance (ANOVA). The ANOVA revealed a significant main effect of group, *F*_(1, 45)_ = 13.70, *p* < 0.001, *f* = 0.26, indicating that thresholds were higher in people with SCZ.

Significant main effects of orientation, *F*_(1, 45)_ = 28.17, *p* < 0.001, *f* = 0.34, and external noise, *F*_(1, 45)_ = 28.27, *p* < 0.001, *f* = 0.38, also were found, indicating higher thresholds were obtained in conditions that used inverted faces and higher levels of external noise. None of the interactions were significant, *F's < 1*, *p* > 0.33, all *f*s = 0.

To use Equation 1 to calculate internal noise for an individual participant, the slope of the noise masking function must be greater than zero (i.e., threshold must be higher in the high-noise condition). Some participants did not display this result and, therefore, internal noise could not be estimated for those subjects. Consequently, subjects who did not have higher noise thresholds in the high-noise condition were removed from the data for further analysis. To minimize the number of subjects that were removed, we applied the criterion (i.e., higher threshold in the high-noise condition) separately in the upright and inverted conditions. This procedure yielded slightly different subsets of subjects in the upright and inverted face conditions, and therefore the two face orientation conditions were analyzed separately.

In conditions using upright faces, data from four participants in the control group and three in the SCZ group were removed. Thresholds from the remaining 20 participants in each group were analyzed with a 2 (group) × 2 (external noise) ANOVA, which revealed significant main effects of group, *F*_(1, 38)_ = 10.73, *p* = 0.002, *f* = 0.35, and external noise level, *F*_(1, 38)_ = 49.48, *p* < 0.001, *f* = 0.55. The group × external noise interaction was not significant, *F*_(1, 38)_ = 0.03, *p* = 0.86, *f* = 0. In conditions using inverted faces, data from five participants in the control group and six participants in the SCZ group were removed. A 2 (group) × 2 (external noise) ANOVA on the remaining subjects found significant main effects of group, *F*_(1, 34)_ = 7.45, *p* = 0.01, *f* = 0.30, external noise, *F*_(1, 34)_ = 63.8, *p* < 0.001, *f* = 0.66; the interaction was not significant, *F*_(1, 34)_ = 0.37, *p* = 0.54, *f* = 0. These analyses indicate that (a) applying the criterion of having a higher threshold in the high-noise condition caused approximately equal numbers of participants to be removed from each group; and (b) the effects of group and external noise measuring in the subsets of subjects were similar to the effects obtained with the entire sample.

Equation 1 was used to estimate equivalent input noise and *k* for each participant who had a higher threshold in the high-noise condition. Figure 7 shows that equivalent input noise did not vary systematically with face orientation: in the control group, equivalent input noise was slightly higher with inverted faces, but in the SCZ group it was slightly lower with inverted faces. In both the upright and inverted face conditions, however, average equivalent input noise was lower in the control group than in the SCZ group. One-tailed *t*-tests performed on log-transformed data indicated that the group difference was significant in the upright face condition, *t*_(38)_ = 2.63, *p* = 0.006, *f* = 0.38, and at trend level significance in the inverted face condition, *t*_(34)_ = 1.41, *p* = 0.08, *f* = 0.16.

**Figure 6:**
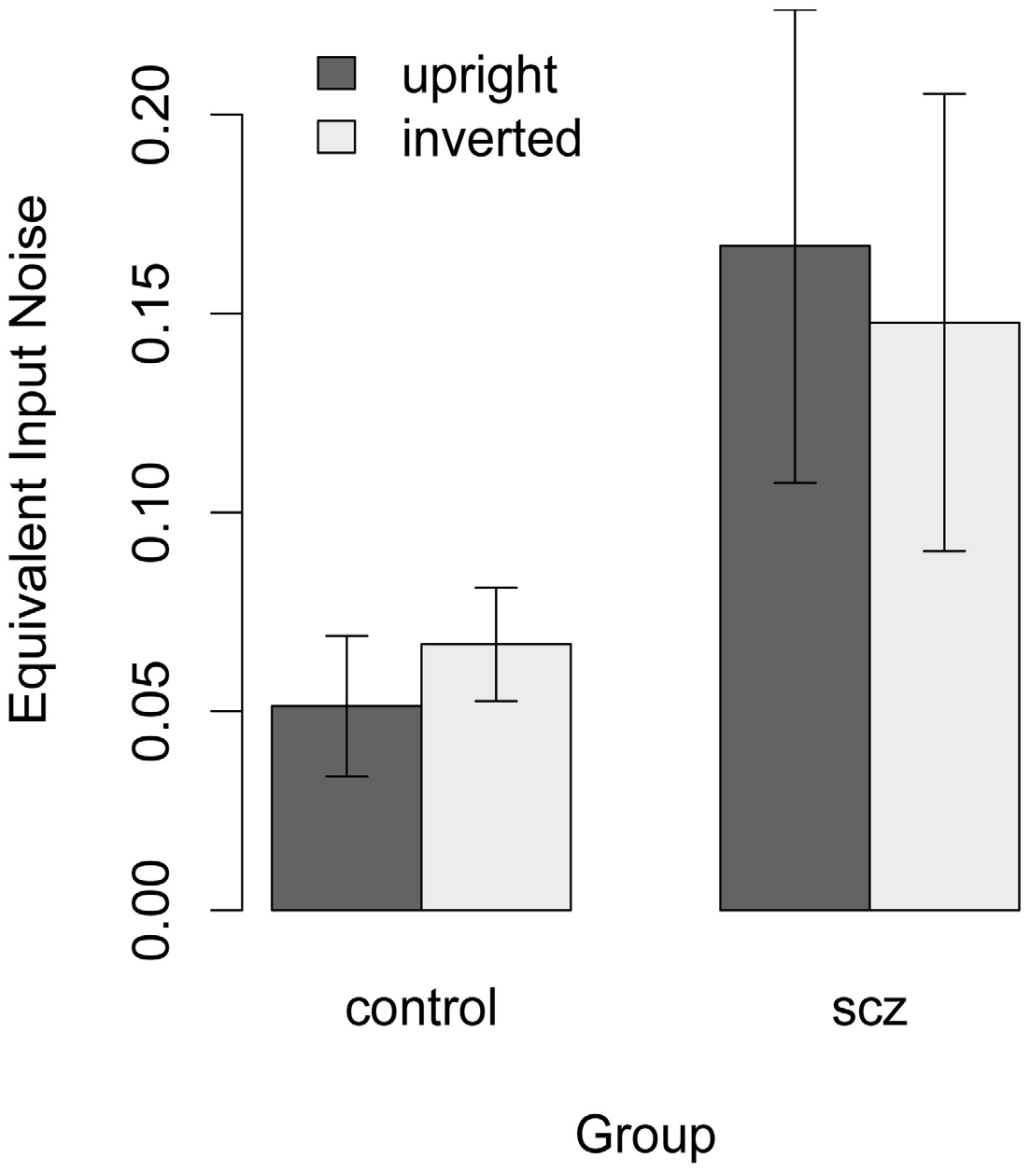
**Estimate of equivalent input noise for each participant who had a higher threshold in the high-noise condition**. For healthy controls equivalent input noise was slightly higher with inverted faces, but in the SCZ group it was slightly lower with inverted faces. One-tailed *t*-tests performed on log-transformed data indicated that the group difference was significant in the upright face condition. Error bars represent ±1 standard error.

Average values of *k* are shown in Figure 6. In both groups, *k* was higher (i.e., calculation efficiency was lower) in the inverted face condition. However, the difference between the control and SCZ groups was not significant with either face orientation [upright: *t*_(38)_ = 0.17, *p* = 0.57, *f* = 0; inverted: *t*_(34)_ = 0.61, *p* = 0.73, *f* = 0].

**Figure 7:**
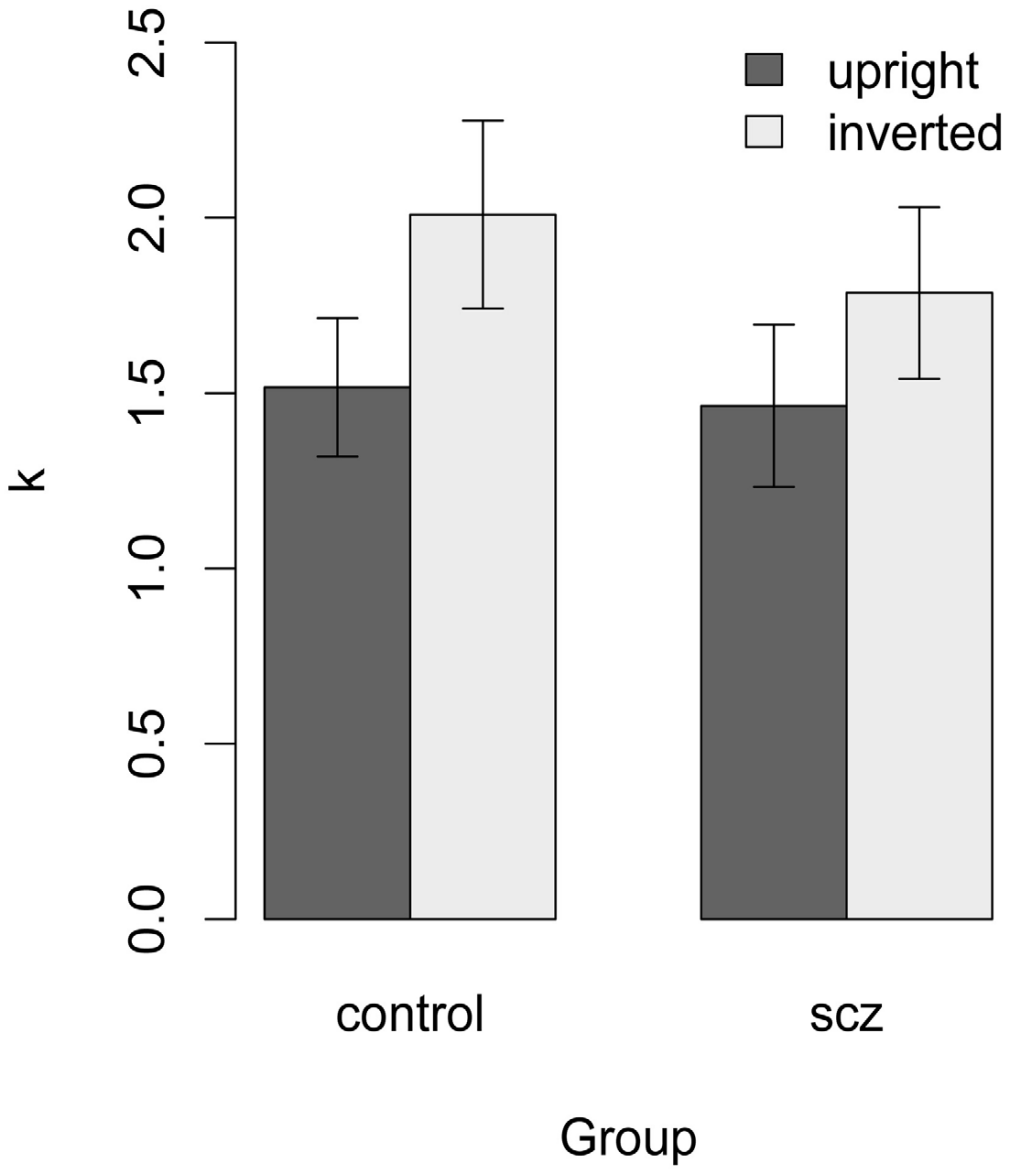
**Estimate of *k* for each participant with a higher threshold in the high-noise condition. In both groups, *k* was higher (i.e., calculation efficiency was lower) in the inverted face condition**. The difference between the control and SCZ groups was not significant with face orientation.

## Discussion

Consistent with previous research, this study found that SCZ participants are deficient in their ability to discriminate both upright and inverted faces compared to healthy observers. However, this study further sought to examine whether this effect was a result of increases in internal noise and/or decreased calculation efficiency. It was found that people with SCZ exhibit higher levels of internal noise when processing both upright and inverted faces; however, it is important to underscore that, while the group differences across the upright and inverted condition were of a similar magnitude, these differences were significant in the upright condition but trended toward significance in the inverted condition. In contrast, no between-group differences were observed on measures of calculation efficiency.

Our results suggest that internal noise is a substantive determinant of face discrimination deficits among people with SCZ. These results are also consistent with increasing evidence that signal-to-noise ratio is decreased among those with SCZ (Winterer and Weinberger, 2000, 2004). Causes of increased internal noise in people with SCZ are unclear. Higher equivalent input noise could be caused by a variety of processes, such as jitter of the eyes or random fluctuations in the responses of sensory neurons. More recent work has suggested that increased noise in those with SCZ may be attributed to an alteration of brain dopamine levels, secondary to reduced NMDA and GABA receptors (Lang et al., 2007).

Although the source of internal noise is unclear, it has been hypothesized by Rolls et al. (2008) that neural noise underlies many of the cognitive and perceptual impairments associated with SCZ. According to the dynamical systems hypothesis, groups of neurons supporting a given behavior or cognition settle into a stable pattern, or attractor state. These attractors are then strengthened over time, and eventually partial activation of the neural network will be sufficient to activate the entire network. However, neural noise (i.e., random spiking) can destabilize the attractor state, and may even induce a sudden change from one attractor state to another, which may result in behavioral distraction or confusion. Rolls et al. (2008) propose that this noise-induced instability of attractor networks may be related to some of the impairments in SCZ, including impairments in memory and attention. While Rolls et al. suggest that the destabilizing of neural networks occurs in the prefrontal cortex, it is possible that the destabilization of neural networks may be a widespread general impairment in SCZ, and may explain much impairment, including face perception, in this population.

As noted above, results also indicate that there are no differences in calculation efficiency between healthy observers and those with SCZ. This is at odds with previously cited scanpath studies showing that people with SCZ direct their voluntary gaze at less informative regions of faces. A plausible explanation for this discrepancy is that scanpath studies actually do not only represent changes in efficiency, but also may be indicative of increased internal noise. In other words, scanpaths may be mediated by attentional processes, and as Rolls et al. (2008) suggest, attention may be deleteriously affected by the destabilization of neural attractor states. Although it is unknown how calculation efficiency and internal noise are related in this context, future research directed at examining this issue would be beneficial.

In the present study, we postulate that a decreased performance in face discrimination among people with SCZ may be a result of an increase in internal noise. It is plausible, however, that increased internal noise is not isolated to face processing, but may also underlie other visual perceptual deficits in SCZ. People with SCZ are also impaired in motion perception (Chen et al., 1999a,b; Clementz et al., 2007), visual context processing (Uhlhaas et al., 2005), and perceptual organization (Silverstein et al., 2006; Sehatpour et al., 2010). The methods as outlined in this study may also be used to disentangle whether increased internal noise contributes to deficiencies in other visual processes. In this context, many studies have demonstrated SCZ-related visual sensory processing deficits on tasks that preferentially involve the magnocellular visual system (Schechter et al., 2003; Butler et al., 2005, 2009; Keri et al., 2005; Martinez et al., 2008). More recently, Butler et al. (2009) investigated magnocellular and parvocellular contributions to emotion processing deficits using affective faces. Butler et al.'s results showed that deficits in low spatial frequency contrast sensitivity, thought to reflect magnocellular visual processing, correlated significantly with the ability to identify emotions from affective faces. Therefore, it is also plausible that SCZ-related performance deficits in affective face processing arise, at least in part, from magnocellular system dysfunction, which, in turn, may reflect greater internal (e.g., neural) noise.

The results from the current study also have clinical implications. As noted above, face processing is deficient among persons with SCZ, which may, in turn, underpin some of the social deficits associated with this disorder. In particular, important cues to others' emotional states and intentions are conveyed via facial expressions and facial affect recognition deficits are prominent features that appear early in the course of the illness and are stable over time (Penn et al., 2008). Deficits in this realm have been demonstrated using instruments such as the Reading the Mind in the Eyes Test (Köther et al., 2012) and the Mayer-Salovey-Caruso Emotional Intelligence Test (Dawson et al., 2012). Accordingly, several social cognitive remediation programs for patients with psychotic disorders have emerged, many of which explicitly train participants to decipher facial expressions as a means to understanding the emotional states of others (for a review see Fiszdon and Reddy, 2012). However, to the extent that such remediation efforts include explicit means for increasing visual focus on informative aspects of the face (e.g., eyes), these efforts may be somewhat misdirected. Although it may be the case that increased attentiveness to informative facial areas will positively influence discrimination or recognition performance across all participants, the current results would not predict disproportionate benefit for persons with SCZ. Instead, methods that may allow one to decipher signals in an otherwise noisy system may be more efficient. For example, sequential sampling models of decision making show that information leading to perceptual or cognitive decisions are quantitatively dependent on the accumulation of information over time via a random-walk process (Ratcliff and McGoon, 2008). In such models, greater noise necessitates greater time to make correct decisions—i.e., the time needed to accumulate adequate information (i.e., drift rate) is longer. Therefore, SCZ-related deficits in face processing may benefit from the simple intervention of encouraging patients to process the information for longer time periods. Similarly, pharmacologic manipulations known to increase neural signal relative to noise may also be a potential avenue for augmenting face processing in this population (Rolls and Deco, 2010).

This study is limited by a modest sample size, following the exclusion of participants necessary for the calculation of internal noise. In this context, replication of the study, in addition to a larger sample size, is essential to confirm these results. Regarding sample characteristics, although participants in the current study were not intentionally matched for education and estimated FSIQ, both healthy controls and people with SCZ demonstrated equivalent results in these domains. This may raise generalizability concerns, as equivalence across these dimensions is not typically observed, and so this patient sample may arguably represent higher functioning participants in the SCZ group. Furthermore, it is unknown whether these findings are specific to SCZ or whether similar results would be observed in other types of psychopathology. It would be useful, therefore, for future studies to explore such potential deficits among persons with a range of psychiatric diagnoses.

Additionally, the current study included the use of only two levels of external noise to determine calculation efficiency and internal noise. The noise masking function, however, could be better represented using multiple levels of external noise in order to obtain more accurate estimations of these measures. Finally, all participants with SCZ who took part in the experiment were medicated and we are unable to comment whether the results seen in the study were confounded by medication status. In this regard, future research would be well advised to include samples of unmedicated SCZ patients.

## Author note

This research was funded by an operating grant to Bruce K. Christensen from The Canadian Psychiatric Research Foundation and by the Canada Research Chair Program (Allison B. Sekuler and Patrick J. Bennett).

### Conflict of interest statement

The authors declare that the research was conducted in the absence of any commercial or financial relationships that could be construed as a potential conflict of interest.

## Footnotes

^1^The study by Chen et al. (2008) is the only experiment known to the authors that directly compares the processing of faces to other objects with similar visual characteristics. It should also be noted, however, that detection accuracy rates from this study show that face processing is more difficult (lower accuracy in both SCZ and HC groups). Consequently, the group by object-type interaction may reflect a difficulty confound (Chapman and Chapman, 1973) and, in turn, could suggest that face processing is inherently more complex.

^2^It is noted that while many studies have shown a reduced face inversion effect among people with SCZ, others (e.g., Chambon et al., 2006; Butler et al., 2008) have shown intact face inversion effects within this population. While the reasons for these discrepancies are unknown, it is possible that methodological differences across studies may account for these findings.

## References

[B1] Archer J. Hay D. C. Young A. W. (1992). Face processing in psychiatric conditions. Br. J. Clin. Psychol. 31, 45–61 10.1111/j.2044-8260.1992.tb00967.x 1559117

[B2] Bennett P. J. Sekuler A. B. Ozin L. (1999). Effects of aging on calculation efficiency and equivalent noise. J. Opt. Soc. Am. A Opt. Image Sci. Vis. 16, 654–668 10069052 10.1364/josaa.16.000654

[B3] Betts L. R. Sekuler A. B. Bennett P. J. (2007). The effects of aging on orientation discrimination. Vision Res. 47, 1769–1780 10.1016/j.visres.2007.02.016 17466355

[B4] Brainard D. H. (1997). The psychophysics toolbox. Spat. Vis. 10, 443–446 10.1163/156856897X00357 9176952

[B5] Brandt J. Benedict R. H. B. (2001). Hopkins Verbal Learning Test–Revised. Odessa, FL: Psychological Assessment Resources

[B8a] Butler P. D. Abeles I. Y. Weiskopf N. G. Tambini A. Jalbrzikowski M. Legatt M. E. (2009). Sensory contributions to impaired emotion processing in schizophrenia. Schizophr. Bull. 35, 1095–1107 10.1093/schbul/sbp109 19793797 PMC2762631

[B7] Butler P. D. Tambini A. Yovel G. Jalbrzikowski M. Ziwich R. Silipo G. (2008). What's in a face. Effects of stimulus duration and inversion on face processing in schizophrenia. Schizophr. Res. 103, 283–292 10.1016/j.schres.2008.03.007 18450426 PMC2755251

[B8] Butler P. D. Zemon V. Schechter I. Saperstein A. M. Hoptman M. J. Lim K. O. (2005). Early-stage visual processing and cortical amplification deficits in schizophrenia. Arch. Gen. Psychiatry 62, 495–504 10.1001/archpsyc.62.5.495 15867102 PMC1298183

[B9a] Chambon V. Baudouin J. Y. Franck N. (2006). The role of configural information in facial emotion recognition in schizophrenia. Neuropsychologia 44, 2437–2444 10.1016/j.neuropsychologia.2006.04.008 16806310

[B9] Chapman L. Chapman J. (1973). Problems in the measurement of cognitive deficits. Psychol. Bull. 79, 380–385 10.1037/h0034541 4707457

[B10] Chen Y. Nakayama K. Levy D. L. Matthysse S. Holzman P. S. (1999a). Psychophysical isolation of a motion-processing deficit in schizophrenics and their relatives and its association with impaired smooth pursuit. Proc. Natl. Acad. Sci. U.S.A. 96, 4724–4729 10.1073/pnas.96.8.4724 10200329 PMC16399

[B11] Chen Y. Palafox G. P. Nakayama K. Levy D. L. Matthysse S. Holzman P. S. (1999b). Motion perception in schizophrenia. Arch. Gen. Psychiatry 56, 149–154 10.1001/archpsyc.56.2.149 10025439

[B12] Chen Y. Norton D. McBain R. Ongur D. Heckers S. (2009). Visual and cognitive processing of face information in schizophrenia: detection, discrimination, and working memory. Schizophr. Res. 107, 92–98 10.1016/j.schres.2008.09.010 18947982 PMC2640943

[B13] Chen Y. Norton D. Ongur D. Heckers S. (2008). Inefficient face detection in schizophrenia. Schizophr. Bull. 34, 367–374 10.1093/schbul/sbm071 17631619 PMC2632392

[B14] Clementz B. A. McDowell J. E. Dobkins K. R. (2007). Compromised speed discrimination among schizophrenia patients when viewing smooth pursuit targets. Schizophr. Res. 95, 61–64 10.1016/j.schres.2007.05.043 17628436 PMC3164492

[B15] Cohen J. (1988). Statistical Power Analysis of the Behavioral Sciences, 2nd Edn. Hillsdale, NJ: Earlbaum Associates

[B16] Dawson S. Kettler L. Burton C. Galletly C. (2012). Do people with schizophrenia lack emotional intelligence. Schizophr. Res. Treat. 2012, 1–8 10.1155/2012/495174 23304499 PMC3530848

[B18] Dosher B. A. Lu Z. (1998). Perceptual learning reflects external noise filtering and internal noise reduction through channel reweighting. Proc. Natl. Acad. Sci. U.S.A. 95, 13988–13993 10.1073/pnas.95.23.13988 9811913 PMC25004

[B18a] Dosher B. A. Lu Z. L. (2000). Mechanisms of perceptual attention in precuing of location. Vision Res. 40, 1269–1292 10.1016/S0042-6989(00)00019-5 10788639

[B19] Eisenberger N. I. (2012). The neural bases of social pain: evidence for shared representations with physical pain. Psychosom. Med. 74, 126–135 10.1097/PSY.0b013e3182464dd1 22286852 PMC3273616

[B21] First M. B. Spitzer R. L. Gibbon M. Williams J. B. W. (2001). Structured Clinical Interview for DSM-IV-TR Axis I Disorders, Research Version. New York, NY: Biometrics Research, New York State Psychiatric Institute

[B21a] Fiszdon J. M. Reddy L. F. (2012). Review of social cognitive treatments for psychosis. Clin. Psychol. Rev. 32, 724–740 10.1016/j.cpr.2012.09.003 23059624

[B22] Freitas-Magalhães A. (2011). “Emotion: from the brain to the face and back,” in Emotional Expression: the Brain and the Face, ed Freitas-Magalhães A. (Oporto: University Fernando Pessoa Press), 1–40

[B23] Gaspar C. Bennett P. J. Sekuler A. B. (2008). The effect of face inversion and contrast-reversal on efficiency and internal noise. Vision Res. 48, 1084–1095 10.1016/j.visres.2007.12.014 18314157

[B24] Gaspar C. M. (2006). The Effects of Stimulus Information and Orientation on Face Processing. Unpublished doctoral dissertation, McMaster University, Hamilton, ON.

[B25] Gobbini M. I. Leibenluft E. Santiago N. Haxby J. V. (2004). Social and emotional attachment in the neural representation of faces. Neuroimage 22, 1628–1635 10.1016/j.neuroimage.2004.03.049 15275919

[B26] Goghari V. M. MacDonald A. W. Sponheim S. R. (2011). Temporal lobe structures and facial emotion recognition in schizophrenia patients and nonpsychotic relatives. Schizophr. Bull. 37, 1281–1294 10.1093/schbul/sbq046 20484523 PMC3196942

[B27] Gold J. Bennett P. J. Sekuler A. B. (1999). Signal but not noise changes with perceptual learning. Nature 402, 176–178 10.1038/46027 10647007

[B28] Gold J. M. Murray R. F. Sekuler A. B. Bennett P. J. Sekuler R. (2005). Visual memory decay is deterministic. Psychol. Sci. 16, 769–774 10.1111/j.1467-9280.2005.01612.x 16181438

[B29] Gold J. M. Sekuler A. B. Bennett P. J. (2004). Characterizing perceptual learning with external noise. Cogn. Sci. 28, 167–207 10.1207/s15516709cog2802_39666987

[B30] Green D. M. Swets J. A. (1966). Signal Detection Theory and Psychophysics. New York, NY: John Wiley and Sons

[B32] Gur R. E. McGrath C. Chan R. M. Schroeder L. Turner T. Turetsky B. I. (2002). An fMRI study of facial emotion processing in patients with schizophrenia. Am. J. Psychiatry 159, 1992–1999 10.1176/appi.ajp.159.12.1992 12450947

[B33] Haxby J. V. Hoffman E. A. Gobbini M. I. (2002). Human neural systems for face recognition and social communication. Biol. Psychiatry 51, 59–67 10.1016/S0006-3223(01)01330-0 11801231

[B34] Highley J. R. McDonald B. Walker M. A. Esiri M. M. Crow T. J. (1999). Schizophrenia and temporal lobe asymmetry. Br. J. Psychiatry. 175, 127–134 10.1192/bjp.175.2.127 10627794

[B37] Kay S. Fiszbein A. Opler L. (1987). The positive and negative syndrome scale (PANSS) for schizophrenia. Schizophr. Bull. 13, 261–276 10.1093/schbul/13.2.261 3616518

[B38] Keil M. (2008). Does face image statistics predict a preferred spatial frequency for human face processing. Proc. Biol. Sci. 275, 2095–2100 10.1098/rspb.2008.0486 18544506 PMC2603213

[B39] Keri S. Kelemen O. Janka Z. Benedek G. (2005). Visual–perceptual dysfunctions are possible endophenotypes of Schizophrenia: evidence from the psychophysical investigation of magnocellular and parvocellular pathways. Neuropsychology 19, 649–656 10.1037/0894-4105.19.5.649 16187883

[B40] Kirk R. E. (1995). Experimental Design: Procedures for the Behavioral Sciences, 3rd Edn. Pacific Grove, CA: Brooks/Cole

[B41] Köther U. Veckenstedt R. Vitzthum F. Roesch-Ely D. Pfueller U. Scheu F. (2012). “Don't give me that look”—overconfidence in false mental state perception in schizophrenia. Psychiatry Res. 30, 1–8 10.1016/j.psychres.2012.03.004 22482796

[B42] Lang U. E. Puls I. Muller D. J. Strutz-Seebohm N. Gallinat J. (2007). Molecular mechanisms of schizophrenia. Cell. Physiol. Biochem. 20, 687–702 10.1159/000110430 17982252

[B43] Lee C. U. Shenton M. E. Salisbury D. F. Kasai K. Onitsuka T. Dickey C. C. (2002). Fusiform gyrus volume reduction in first-episode schizophrenia. Arch. Gen. Psychiatry 59, 775–781 10.1001/archpsyc.59.9.775 12215076

[B44] Legge G. Kersten D. Burgess A. E. (1987). Contrast discrimination in noise. J. Opt. Soc. Am. A 4, 391–406 10.1364/JOSAA.4.000391 3559785

[B44a] Little A. C. Jones B. C. DeBruine L. M. (2011). Facial attractiveness: evolutionary based research. Phil. Trans. R. Soc. B 366, 1638–1659 10.1098/rstb.2010.0404 21536551 PMC3130383

[B45] Martinez A. Hillyard S. A. Dias E. C. Hagler D. J. Butler P. D. Guilfoyle D. M. (2008). Magnocellular pathway impairment in Schizophrenia: evidence from functional magnetic resonance imaging. J. Neurosci. 28, 7492–7500 10.1523/JNEUROSCI.1852-08.2008 18650327 PMC6670855

[B46] Marwick K. Hall J. (2008). Social cognition in schziohprenia: a review of face processing. Br. Med. Bull. 88, 43–58 10.1093/bmb/ldn035 18812413

[B47] Morey L. C. (1991). Personality Assessment Inventory. Odessa, FL: Psychological Assessment Resources

[B48] Novic J. Luchins D. Perline R. (1984). Facial affect recognition in schizophrenia. Is there a differential deficit? Br. J. Psychiatry. 144, 533–537 10.1192/bjp.144.5.533 6733380

[B49] Onitsuka T. Shenton M. E. Kasai K. Nestor P. G. Toner S. K. Kikinis R. (2003). Fusiform gyrus volume reduction and facial recognition in chronic schizophrenia. Arch. Gen. Psychiatry 60, 349–355 10.1001/archpsyc.60.4.349 12695311

[B50] Pelli D. G. (1997). The VideoToolbox software for visual psychophysics: transforming numbers into movies. Spat. Vis. 10, 437–442 10.1163/156856897X00366 9176953

[B51] Pelli D. G. (1981). Effects of Visual Noise. Unpublished doctoral thesis, University of Cambridge, Cambridge, UK.

[B52] Pelli D. G. Farell B. (1999). Why use noise. J. Opt. Soc. Am. A Opt. Image Sci. Vis. 16, 647–653 10.1364/JOSAA.16.000647 10069051

[B53] Penn D. L. Corrigan P. W. Bentall R. P. Racenstein J. M. Newman L. (1997). Social cognition in schizophrenia. Psychol. Bull. 121, 114–132 10.1037/0033-2909.121.1.114 9000894

[B54] Penn D. L. Sanna L. J. Roberts D. L. (2008). Social cognition in schizophrenia: an overview. Schizophr. Bull. 34, 408–411 10.1093/schbul/sbn014 18375928 PMC2632430

[B55] Phillips M. David A. (1995). Facial processing in schizophrenia and delusional misidentification: cognitive neuropsychiatric approaches. Schizophr. Res. 17, 109–114 10.1016/0920-9964(95)00035-K 8541243

[B56] Quinn K. A. Macrae C. N. (2011). The face and person perception: insights from social cognition. Br. J. Psychol. 102, 849–867 10.1111/j.2044-8295.2011.02030.x 21988388

[B57] Quinn K. A. Macrae C. N. Bodenhausen G. V. (2003). “Stereotyping and impression formation,” in SAGE Handbook of Social Psychology, eds Hogg M. A. Cooper J. (Thousand Oaks, CA: SAGE Publications), 87–109

[B58] Ratcliff R. McGoon G. (2008). The diffusion decision model: theory and data for two-choice decision tasks. Neural Comput. 20, 873–922 10.1162/neco.2008.12-06-420 18085991 PMC2474742

[B59] R Development Core Team. (2007). R: A Language and Environment for Statistical Computing. Vienna: R Foundation for Statistical Computing

[B60] Rolls E. T. Deco G. (2010). The Noisy Brain: Stochastic Dynamics as a Principle of Brain Function. London: Oxford University Press 10.1016/j.pneurobio.2009.01.006 19428958

[B61] Rolls E. T. Loh M. Deco G. Winterer G. (2008). Computational models of schizophrenia and dopamine modulation in the prefrontal cortex. Nat. Rev. Neurosci. 9, 696–709 10.1038/nrn2462 18714326

[B62] Sachs G. Steger-Wuchse D. Kryspin-Exner I. Gur R. C. Katschnig H. (2004). Facial recognition deficits and cognition in schizophrenia. Schizophr. Res. 68, 27–35 10.1016/S0920-9964(03)00131-2 15037337

[B63] Schechter I. Butler P. D. Silipo S. Zemon V. Javitt D. C. (2003). Magnocellular and parvocellular contributions to backward masking dysfunction in schizophrenia. Schizophr. Res. 64, 91–101 10.1016/S0920-9964(03)00008-2 14613674

[B65] Sehatpour P. Dias E. C. Butler P. D. Revheim N. Guilfoyle D. N. Foxe J. J. (2010). Impaired visual object processing across an occipital-frontal-hippocampal brain network in schizophrenia: an integrated neuroimaging study. Arch. Gen. Psychiatry 67, 772–782 10.1001/archgenpsychiatry.2010.85 20679585 PMC4283949

[B66] Sekuler A. B. Gaspar C. M. Gold J. M. Bennett P. J. (2004). Inversion leads to quantitative, not qualitative, changes in face processing. Curr. Biol. 14, 391–396 10.1016/j.cub.2004.02.028 15028214

[B68] Silverstein S. M. Hatashita-Wong M. Schenkel L. S. Wilkniss S. Kovács I. Fehér A. (2006). Reduced top-down influences in contour detection in schizophrenia. Cogn. Neuropsychiatry 11, 112–132 10.1080/13546800444000209 16537237

[B70] Swets J. A. (1996). Signal Detection Theory and ROC Analysis in Psychology and Diagnostics: Collected Papers. Mahway, NJ: Lawrence Earlbaum Associates

[B71] Tjan B. S. Braje W. L. Legge G. E. Kersten D. (1995). Human efficiency for recognizing 3-D objects in luminance noise. Vision Res. 35, 3053–3069 10.1016/0042-6989(95)00070-G 8533342

[B73] Tulloch A. D. Fearon P. David A. S. (2006). Social outcomes in schizophrenia: from description to action. Curr. Opin. Psychiatry 19, 140–144 10.1097/01.yco.0000214338.29378.29 16612193

[B74] Uhlhaas P. J. Phillips W. A. Silverstein S. M. (2005). The course and clinical correlates of dysfunctions in visual perceptual organization in schizophrenia during the remission of psychotic symptoms. Schizophr. Res. 75, 183–192 10.1016/j.schres.2004.11.005 15885509

[B76] Walker E. McGuire M. Bettes B. (1984). Recognition and identification of facial stimuli by schizophrenics and patients with affective disorders. Br. J. Clin. Psychol. 23, 37–44 10.1111/j.2044-8260.1984.tb00624.x 6697027

[B77] Wang S. S. Moon S. I. Kwon K. H. Evans C. A. Stefanone M. A. (2010). Face off: implications of visual cues on initiating friendship on facebook. Comput. Hum. Behav. 26, 226–234 10.1016/j.chb.2009.10.001

[B78] Wechsler D. (1997). Wechsler Adult Intelligence Scale, 3rd Edn. San Antonio, TX: The Psychological Corporation

[B79] Whittaker J. F. Deakin J. F. Tomenson B. (2001). Face processing in schizophrenia. Psychol. Med. 31, 499–507 10.1017/S0033291701003701 11305858

[B80] Wilkinson G. S. (1993). Wide Range Achievement Test, 3rd Edn. Wilmington, DE: Wide Range, Inc

[B81] Williams L. M. Loughland C. M. Gordon E. Davidson D. (1999). Visual scanpaths in schizophrenia: is there a deficit in face recognition. Schizophr. Res. 40, 189–199 10.1016/S0920-9964(99)00056-0 10638857

[B82a] Winterer G. Weinberger D. R. (2004). Genes, dopamine and cortical signal-to-noise ratio is schizophrenia. Trends Neurosci. 27, 683–690 10.1016/j.tins.2004.08.002 15474169

[B82] Winterer G. Ziller M. Dorn H. Frick K. Mulert C. Wuebben Y. (2000). Schizophrenia: reduced signal-to-noise ratio and impaired phase-locking during information processing. Clin. Neurophysiol. 111, 837–849 10.1016/S1388-2457(99)00322-3 10802455

[B84] Yoon J. H. D'Esposito M. Carter C. S. (2006). Preserved function of the fusiform face area in schizophrenia as revealed by fMRI. Psychiatry Res. 148, 205–216 10.1016/j.pscychresns.2006.06.002 17095198

